# Circular RNA F-circEA-2a expression is increased in gastric adenocarcinoma and inhibits the transition from premature microRNA-3940-5p to mature microRNA-3940-5p

**DOI:** 10.1080/21655979.2022.2038935

**Published:** 2022-03-02

**Authors:** Bo Hu, Fei Xiao

**Affiliations:** Department of Gastrointestinal and Anorectal Surgery, Wuhan Fourth Hospital, Wuhan Orthopaedic Hospital, Puai Hospital, Tongji Medical College, Huazhong University of Science and Technology, West Hospital District, Wuhan, Hubei Province, China

**Keywords:** Gastric adenocarcinoma, miR-3940-5p, F-circEA-2a, maturation, proliferation

## Abstract

Circular RNA (circRNA) F-circEA-2a and micorRNA (miR)-3940-5p are two non-coding RNAs with critical roles in cancer biology. However, their participation in gastric adenocarcinoma (GA) is unclear. We predicted that miR-3940-5p could bind to F-circEA-2a and speculated that miR-3940-5p may interact with F-circEA-2a to participate in cancer biology. This study was conducted to explore the interaction between F-circEA-2a and miR-3940-5p in GA. F-circEA-2a and miR-3940-5p (mature and premature) levels in GA were detected using RT-qPCR. Their correlations were analyzed by Pearson’s correlation coefficient. The role of F-circEA-2a in miR-3940-5p maturation was analyzed using overexpression assay. The direct binding of premature miR-3940-5p to F-circEA-2a was analyzed by RNA-RNA pulldown. Proliferation was analyzed with BrdU assay. We found that F-circEA-2a and premature miR-3940-5p were overexpressed in GA, while mature miR-3940-5p was under-expressed in GA. F-circEA-2a suppressed miR-3940-5p maturation in GA cells. MiR-3940-5p directly bound to F-circEA-2a wild type (-wt), but not mutant (-mut). F-circEA-2a promoted GA cell proliferation and inhibited the role of miR-3940-5p in reducing cell proliferation. Therefore, F-circEA-2a might suppress mature miR-3940-5p formation by sponging premature miR-3940-5p to promote cell proliferation in GA. Our study characterized a novel circRNA regulating miR-3940-5p maturation in GA.

## Graphical Abstract

**Figure:**
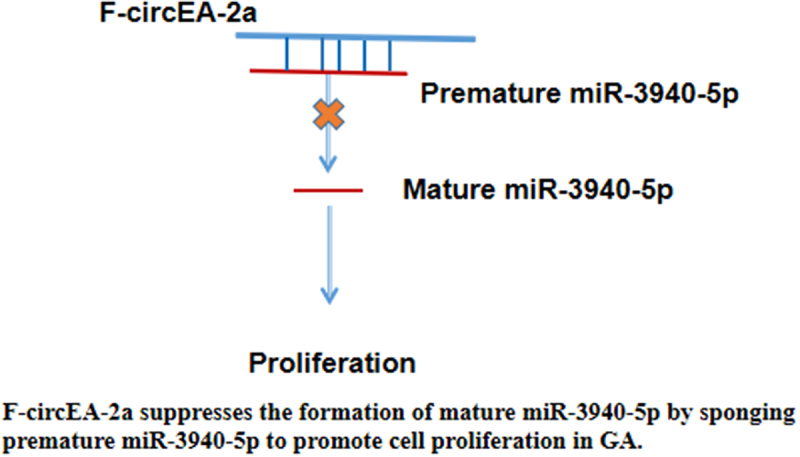

## Introduction

Gastric cancer is a solid, malignant tumor originating from the cells in the stomach lining [1]. As a severe health problem worldwide, gastric cancer accounts for about 90% gastric adenocarcinomas (GA) and affects more than 1 million patients each year [2]. Infection of *Helicobacter pylori* is the cause of most GA cases [3,4]. It is estimated that more than 47% of Chinese in urban area and more than 66% of Chinese in rural area are *H. pylori* positive [5]. As a consequence, China accounts for more than 45% of global GA cases [6]. However, to date, there is no cure for most cases of GA, especially for those with metastatic tumors [7].

At present, total/subtotal gastrectomy or endoscopic mucosal resection can only be applied to GA patients at early stages [8]. Surgical approaches are not appropriate for metastatic patients. Although chemotherapy or radiotherapy can be used to treat metastatic GA, these approaches can only care for the symptoms and lengthen patients’ life [9]. In addition, chemotherapy and radiotherapy can cause side effects in a considerable portion of patients. Even worse, recurrence even after surgical resection is common, leading to poor prognosis [8,9]. Due to the complexity of cancer development, cure is rare, and the development of adverse effects is common [10]. In recent years, molecular mechanism of GA has been rapidly elucidated and some molecular factors are likely to be targets in targeted therapy to treat GA [11,12]. However, more targets are needed in research and clinical trials to select the most promising ones.

Circular RNAs (circRNAs) have limited or even no coding information. But they can interact with DNAs, proteins, and other RNAs to impact cancers, suggesting their potentials as targets to treat GA [13,14]. CircRNA F-circEA-2a and microRNA (miR)-3940-5p are two recently identified RNAs with critical roles in cancer biology [15,16]. F-circEA-2a has been reported to promote lung cancer cell migration and invasion [15]. In contrast, miR-3940-5p suppresses lung cancer progression by downregulating multiple oncogenes, such as ubiquitin-specific peptidase-28 and cyclin D1 [16]. However, their participation in GA is unclear. We predicted that miR-3940-5p could bind to F-circEA-2a and speculated that miR-3940-5p and F-circEA-2a may interact with each other to participate in cancer biology, such as GA. Therefore, we studied the interaction between F-circEA-2a and miR-3940-5p in GA and found that F-circEA-2a could suppress miR-3940-5p maturation by sponging premature miR-3940-5p to increase GA cell proliferation. Our study identified a novel circRNA regulator in miR-3940-5p maturation and suggested the F-circEA-2a/miR-3940-5p axis as clinical target for GA diagnosis and treatment. Together with the recent advances in delivering RNA molecules with therapeutic effects into tumor cells [17–19], more effectively treatment approaches for cancers may be developed.

## Materials and methods

### Clinical samples

This research enrolled 70 GA patients (32 females and 38 males at an average age of 56.7±±7.2 years; 31 cases of stage I/II and 39 cases of stage III/IV) at Wuhan Fourth Hospital; Puai Hospital, Tongji Medical College, Huazhong University of Science and Technology after Ethics approval was obtained from the Ethics Committee of this hospital and informed consent was obtained. GA patients diagnosed with biopsy during upper endoscopy and without initiation of therapy within 3 months prior to admission were included. Patients with recurrent diseases, complicated with other severe diseases, and blood relationship were excluded. Resected tumors (n = 58) and biopsy samples (n = 12) were collected and pathologically analyzed to separate GA and paired non-tumor samples.

### Cells and culture methods

*In vitro* experiments were performed using HGC-27 and MKN-45 cell lines (ATCC). Subculture was performed at about 70–80% confluency. Briefly, cells were cultured in RPMI-1640 (Catalog # 11875093, Thermo Fisher) containing 10% FBS (Catalog # F2442-50ML, Sigma-Aldrich) in 6-cm plates. Contamination was monitored daily, and cells were discarded if contamination was observed.

### Transfection assays

To study the function of genes and gene interactions, transient cell transfection was conducted using lipofectamine 3000 (Invitrogen). Briefly, cells were collected, washed with PBS three times, and counted. About 10^8^ cells were incubated 6 h with the transfection mixture, which was prepared by mixing lipofectamine 3000 (Catalog # L3000001, Thermo Fisher) with medium containing 10 nM F-circEA-2a vector or 50 nM miR-3940-5p mimic. After incubation, cells were collected, washed three times with fresh medium, transferred to a 6-cm plate containing 15 ml fresh medium, and cultured for 48 h prior to the subsequent experiments.

### RNA preparations

To isolate RNA with high quality, samples were mixed with RNAzol (Cat# R4533, Sigma-Aldrich) at 10:1 ratio (volume) and incubated at room temperature for 40 min. The mixtures were centrifuged at 12,000 g for 10 min to collect the supernatants. The supernatants were mixed with chloroform at 4:1 ratio (volume) and centrifuged at 12,000 g for 10 min. The supernatants were collected, mixed with methanol at 1:1 ratio (volume), and centrifuged at 12,000 g for 10 min. The RNAs in the supernatants were precipitated, washed three times with 70% ethanol, and resuspended in nuclease-free water.

### RNA analysis and RT-qPCR

RNA purity, quality, and quantity were determined using a 2100 bioanalyzer (Agilent). RNA samples with satisfactory parameters were prepared as cDNA samples using SSRT IV system (Cat# 18091050, Thermo Fisher) after addition of poly(A) tail. All cDNA samples were digested with RNase H (included in SSRT IV kit) and subjected to PCR amplification of GAPDH to determine cDNA quality. All cDNA samples with satisfactory quality were subjected to qPCR using SYBR® Green Quantitative RT-qPCR Kit (Cat# QR0100-1KT, Sigma-Aldrich) to determine F-circEA-2a and miR-3940-5p levels with 18S rRNA as the internal control. Gene expression was normalized using the 2^−ΔΔCt^ method [20]. Primer sequences were 5’-CAACTACTGCTTTGCTGGCA-3’ (forward) and 5’-TGCTCGAATTCAGAGCACAC-3’ (reverse) for F-circEA-2a; 5’-ACCACAGTCCATGCCATCAC-3’ (forward) and 5’-TCCACCACCCTGTTGCTGTA-3’ (reverse) for GAPDH; 5’-GCTTATCGAGGAAAAGATCGAGG-3’ (forward) and 5’-GAAGGAAGGAGAGTAACCAAGGTA-3’ (reverse) for premature miR-3940-5p; and 5’-GTGGGTTGGGGCGGGCT-3’ (forward) and poly(T) for mature miR-3940-5p.

### RNA-RNA pulldown assay

The direct interaction between F-circEA-2a and miR-3940-5p was analyzed by performing RNA-RNA pulldown assay using biotin-labeled F-circEA-2a wild type (Bio-F-circEA-2a-wt), F-circEA-2a mutant (Bio-F-circEA-2a-mut), and NC (Bio-NC), which were prepared via *in vitro* transcription with T7 RNA polymerase (Cat# M0251S, NEB) and biotin labeling (Biotin RNA Labeling Mix, Cat# 11685597910, Sigma-Aldrich). Cells were transfected with RNAs and collected 48 h later. Then, cells were lysed, and RNA complexes were pulled down using streptavidin-coated magnetic beads (Cat# 88816, Thermo Fisher). Then, the accumulation of premature miR-3940-5p in pulldown samples was determined using RT-qPCR.

### BrdU assay

Cell proliferation was analyzed by determining BrdU incorporation [21] using the BrdU Cell Proliferation ELISA Kit (Cat# ab126556, Abcam). In brief, transfected cells were collected and washed with PBS three times. The cells were then transferred to 24-well plates with three replicates in each experiment. Cells were cultured for 48 h and incubated with BrdU (10 mg). After 2 h, cells were incubated first with anti-BrdU antibody for 2 h, then with of tetramethylbenzidine, the substrate of the antibody. Finally, OD values at 450 nm were determined, and cell proliferation was normalized to the negative controls, which were incubated with BrdU and etramethylbenzidine but not anti-BrdU antibody.

### Statistical analysis

All data and images were prepared using GraphPad Prism 8. Differences between two and among more than two groups were compared using ANOVA Tukey's test and Student’s *t*-test, respectively. A p value smaller than 0.5 was considered statistically significant.

## Results

### Accumulation of F-circEA-2a and miR-3940-5p (mature and premature) in GA

**Figure 1.**
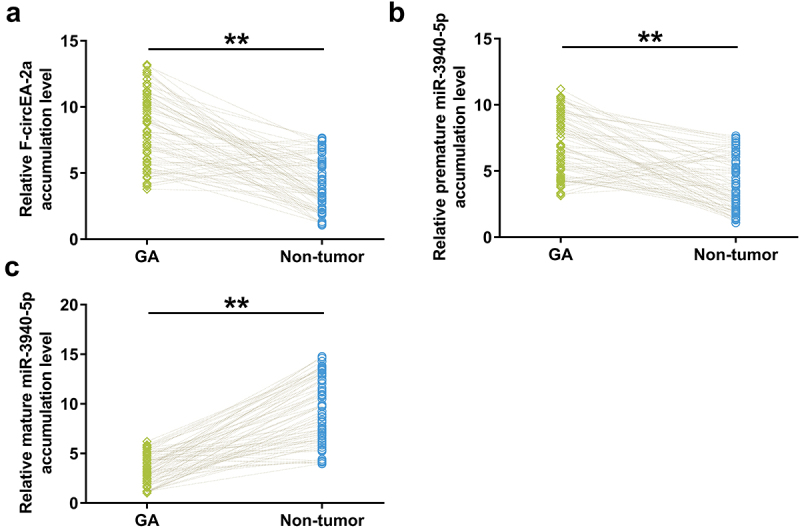
Accumulation of F-circEA-2a and miR-3940-5p (mature and premature) in GA Accumulation of F-circEA-2a, premature miR-3940-5p, and mature miR-3940-5p in both GA and non-tumor samples from GA patients (n = 70) was analyzed using RT-qPCR. Average values of three technical qPCR replicates were presented and compared. **, p < 0.01.

Accumulation of F-circEA-2a, and premature and mature miR-3940-5p in both GA and non-tumor samples from GA patients (n = 70) were analyzed using RT-qPCR to explore their potential involvement in GA. The data showed that F-circEA-2a (Figure 1(a), p < 0.01) and premature miR-3940-5p (Figure 1(b), p < 0.01) were overexpressed in GA, while mature miR-3940-5p was under-expressed in GA (Figure 1(c), p < 0.01). Therefore, decreased F-circEA-2a accumulation and miR-3940-5p maturation may contribute to GA development or the consequences of GA.

### Correlation analysis between F-circEA-2a and miR-3940-5p (mature and premature)

**Figure 2.**
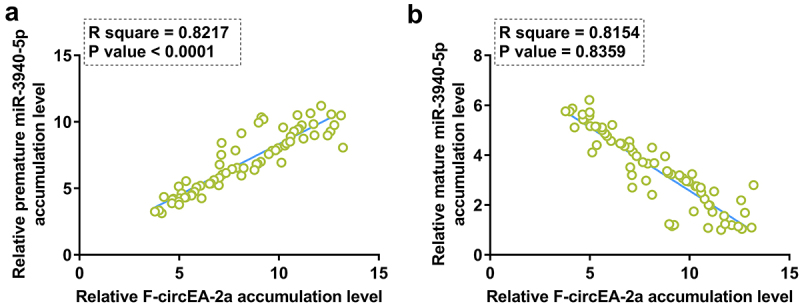
Correlation analysis between F-circEA-2a and miR-3940-5p (mature and premature) The correlation of F-circEA-2a with premature miR-3940-5p (a) or mature miR-3940-5p (b) was analyzed by Pearson’s correlation coefficient. Data presented in Figure 1 was used in this analysis.

Correlation analysis may provide insights into functional analysis. To this end, the correlation of F-circEA-2a with premature miR-3940-5p or mature miR-3940-5p was analyzed by Pearson’s correlation coefficient. Interestingly, F-circEA-2a showed a positive correlation with premature miR-3940-5p (Figure 2(a)) but a negative correlation with mature miR-3940-5p (Figure 2(b)). Therefore, F-circEA-2a may be involved in miR-3940-5p maturation.

### Binding of premature miR-3940-5p to F-circEA-2a

**Figure 3.**
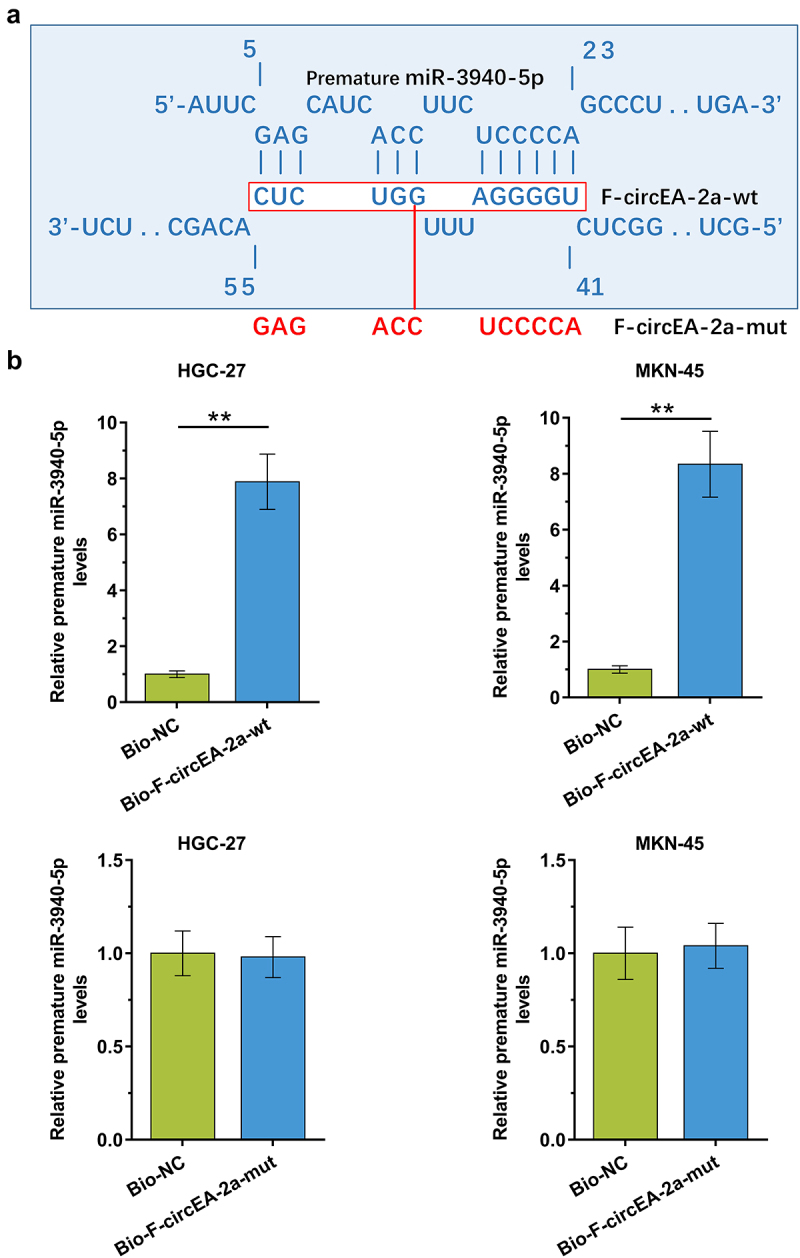
The binding of premature miR-3940-5p to F-circEA-2a The binding of premature miR-3940-5p to F-circEA-2a was predicted by IntaRNA 2.0 (a). The binding of premature miR-3940-5p to F-circEA-2a-wt (b) and F-circEA-2a-mut (B), which was labeled with red color in (A), was confirmed by RNA-RNA pull-down assay using biotin (Bio)-labeled RNAs. The enrichment of premature miR-3940-5p suggested the direct interaction between the two molecules. Data presented are the mean ± SD values of three biological replicates. **, p < 0.01.

Direct interaction suggests function. To this end, the binding of premature miR-3940-5p to F-circEA-2a was predicted by IntaRNA 2.0. The prediction showed that premature miR-3940-5p may bind to F-circEA-2a (Figure 3(a)). Furthermore, the binding of premature miR-3940-5p to F-circEA-2a wild type (F-circEA-2a-wt) and mutant (F-circEA-2a-mut, labeled with red color Figure 3(a)) in HGC-27 and MKN-45 cells was analyzed using RNA-RNA pulldown assay. The results confirmed that premature miR-3940-5p directly bound to F-circEA-2a-wt (Figure 3(b), p < 0.01) but not F-circEA-2a-mut (Figure 3(c), p < 0.01). Therefore, F-circEA-2a can directly interact with premature miR-3940-5p in GA cells.

### Regulation of miR-3940-5p maturation by F-circEA-2a

**Figure 4.**
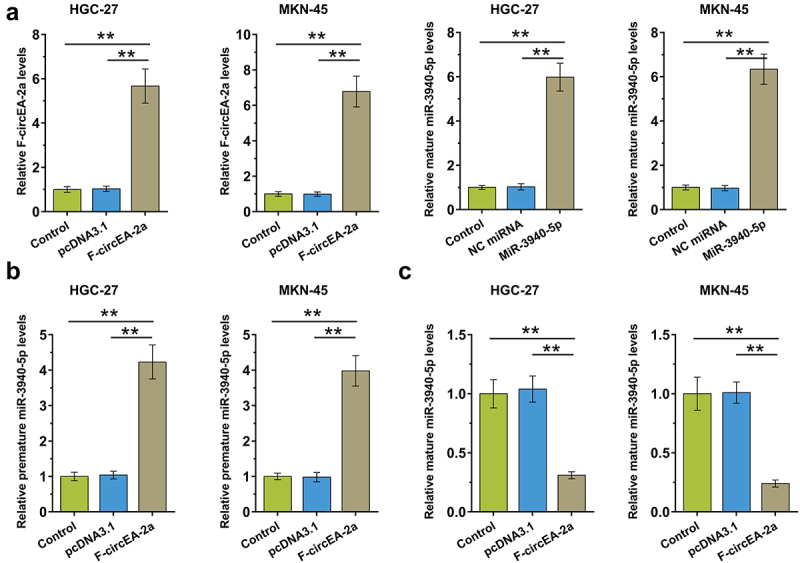
Regulation of miR-3940-5p maturation by F-circEA-2a HGC-27 and MKN-45 cells were overexpressed with F-circEA-2a and miR-3940-5p to explore their interaction (a). The role of F-circEA-2a in premature miR-3940-5p (b) and mature miR-3940-5p (c) accumulation was explored by RT-qPCR. Data presented are the mean ± SD values of three biological replicates. **, p < 0.01.

To study the role of F-circEA-2a in miR-3940-5p maturation, HGC-27 and MKN-45 cells were overexpressed with F-circEA-2a and miR-3940-5p to explore their interaction (Figure 4(a), p < 0.01). RT-qPCR showed that F-circEA-2a increased premature miR-3940-5p accumulation (Figure 4(b), p < 0.01) but decreased mature miR-3940-5p accumulation (Figure 4(c), p < 0.01). Therefore, F-circEA-2a can suppress miR-3940-5p maturation in GA cells.

### Regulation of HGC-27 and MKN-45 cell proliferation by miR-3940-5p and F-circEA-2a

**Figure 5.**
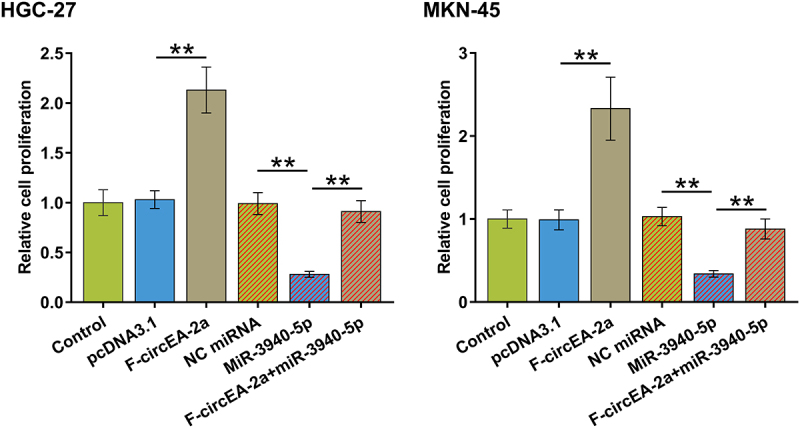
Regulation of HGC-27 and MKN-45 cell proliferation by miR-3940-5p and F-circEA-2a Regulation of HGC-27 and MKN-45 cell proliferation by miR-3940-5p and F-circEA-2a was explored by BrdU assay. Data presented are the mean ± SD values of three biological replicates. **, p < 0.01.

To further explore the role of interaction between miR-3940-5p and F-circEA-2a in regulating HGC-27 and MKN-45 cell proliferation, we performed BrdU assay and found that F-circEA-2a promoted while miR-3940-5p suppressed both cell proliferation. Interestingly, F-circEA-2a attenuated the inhibitory effect of miR-3940-5p on cell proliferation (Figure 5, p < 0.01). Therefore, F-circEA-2a may promote GA cell proliferation through miR-3940-5p.

## Discussion

MiRNA maturation determines the functional achievement of miRNAs. This research studied the involvement of miR-3940-5p and F-circEA-2a in GA, with a focus on the regulatory role of F-circEA-2a in miR-3940-5p maturation. We characterized F-circEA-2a as an upstream inhibitor of miR-3940-5p in cancer biology.

The progression of lung cancer is correlated with altered F-circEA-2a accumulation in cancer tissues [15]. F-circEA-2a accumulation is increased in the cytoplasm of lung cancer cells, resulting in increased cell invasion. However, the mechanism underlying the function of F-circEA-2a in lung cancer is unclear. The present research reported the increased F-circEA-2a accumulation in GA tissues. In two GA cell lines, F-circEA-2a overexpression increases cell proliferation. Therefore, F-circEA-2a likely plays an oncogenic role in GA by regulating cancer cell proliferation.

MiR-3940-5p has been characterized as a tumor suppressor in many cancers [16]. The development of lung cancer is related to decreased miR-3940-5p accumulation in cancer cells via targeting ubiquitin-specific peptidase 28 and cyclin D1 to suppress cancer progression. We observed the decreased miR-3940-5p accumulation in GA and its inhibitory effects on cancer cell proliferation in GA. Therefore, miR-3940-5p inhibits GA development.

Although the role of miR-3940-5p in many cancers has been explored, its upstream regulators are largely unknown. This research reported the inhibitory effects of F-circEA-2a on miR-3940-5p maturation in two GA cell lines. Moreover, premature miR-3940-5p in both cell lines could bind to wild-type F-circEA-2a but not F-circEA-2a mutant with disrupted miR-3940-5p binding sites. Therefore, F-circEA-2a can directly interact with premature miR-3940-5p in the nucleus. It has been well established that premature miRNAs movement from the nucleus to cytoplasm is the most critical step for miRNAs maturation. Therefore, F-circEA-2a may bind to premature miR-3940-5p in the nucleus to reduce its transportation from the nucleus, leading to decreased mature miR-3940-5p production. Different from previous studies that mainly focus on the role of circRNAs as competing RNAs for mature miRNAs [22–25], our study reports the regulatory role of a circRNA in miRNA maturation via direct sponging its premature form. The proposed novel interaction between lncRNAs and miRNAs may guide future studies of lncRNAs and their interactions with miRNAs.

## Conclusion

F-circEA-2a may suppress mature miR-3940-5p formation by sponging premature miR-3940-5p to promote cell proliferation in GA. Our study characterized a novel F-circEA-2a/miR-3940-5p axis in GA. Future studies may explore the application of this novel pathway in the treatment of GA, with a focus on the techniques to deliver RNAs to tumor cells.

## Data Availability

The datasets used and/or analyzed during the current study are available from the corresponding author on reasonable request.
